# Cerebral aspergillosis presenting as a space occupying lesion in an immunocompetent individual

**DOI:** 10.1016/j.mmcr.2019.07.011

**Published:** 2019-08-01

**Authors:** Georges El Hasbani, Joseph Chirayil, Sutasinee Nithisoontorn, Arturo Alvarez Antezana, Ibrahim El Husseini, Maria Landaeta, Yasir Saeed, Richard Assaker

**Affiliations:** a American University of Beirut, Beirut, Lebanon; b Lincoln Medical and Mental Health Center, Bronx, New York, USA; c St. Vincent's Medical Center, Bridgeport, CT, USA

**Keywords:** Cerebral aspergillosis, Immunocompetence, Neuroimaging

## Abstract

Cerebral aspergillosis has the tendency to occur in immunocompromised patients. Less commonly, immunocompetent individuals can be affected, with neuroimaging findings being difficult to interpret. The diagnosis necessitates imaging of the brain as well as the sinuses with biopsy and pathological confirmation. A surgical excision with aggressive antifungal agents are required for a proper management. This case report describes an immunocompetent patient with cerebral aspergillosis that presented radiologically as a suspicious mass to be diagnosed pathologically and excised surgically.

## 1 Introduction

Aspergillosis is a tissue infection caused by fungi of the genus *Aspergillus*. Cerebral aspergillosis is a very rare entity that has a high mortality rate [1]. *Aspergillus* reaches the brain most likely through hematogenous dissemination from the lungs although other mechanisms exist [2]. Despite that immunocompromised patients are the target population, several case reports have described the occurrence of invasive aspergillosis in apparently immunocompetent individuals. The classical neuroimaging finding of CNS aspergillosis described in immunocompetent patients as a mass with thick irregular walls is not often observed in immunocompromised individuals making the diagnosis a difficult one [3]. This case report describes an immunocompetent person who presented for sudden onset unresponsiveness. His brain imaging was suspicious for a malignant intracerebral mass extending to the nasal cavity. Imaging of the sinuses with biopsy confirmed the diagnosis of cerebral aspergillosis. Excision of the mass with surgical debridement yielded a good outcome.

## 2 Case

A 56 year-old male with a history of left eye blindness due to untreated glaucoma, hypertension, and diabetes mellitus type 1 presented to the emergency department with sudden onset unresponsiveness. The son, who witnessed the event, reported that the patient suddenly became rigid, still, and unresponsive. He had labored breathing for 5 minutes. The patient returned to baseline in 20 minutes. There was no prior history of similar symptoms. The patient was working in a transporting grocery store. He used to work as a coal miner 10 years before presentation.

On examination, the patient appeared comfortable, and was alert and oriented. He had a contracted fifth finger along with an old surgical scar on the right forehead, anisocoria with slight left facial deviation. The remaining examination was within normal limits. His complete blood count, comprehensive metabolic panel, and EKG were all normal except for a hemoglobin A1C level of 9.7%.

**Fig. 1:**
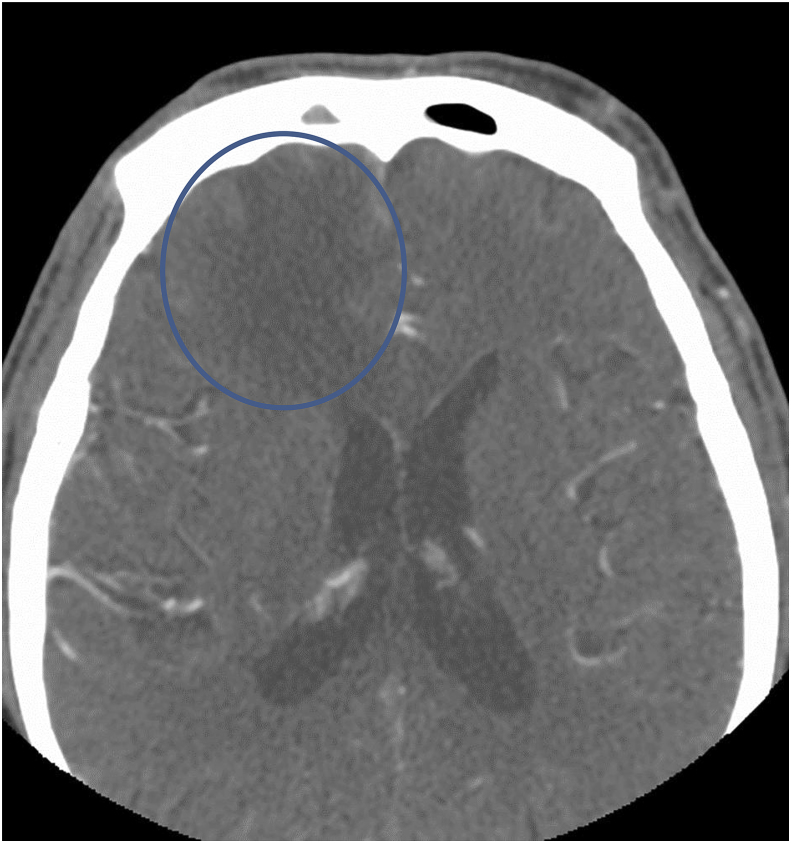
Axial CT scan of the brain with IV contrast showing vasogenic edema within the right frontal corona radiata and centrum semiovale. Mild mass effect on the frontal horns of both lateral ventricles.

**Fig. 2:**
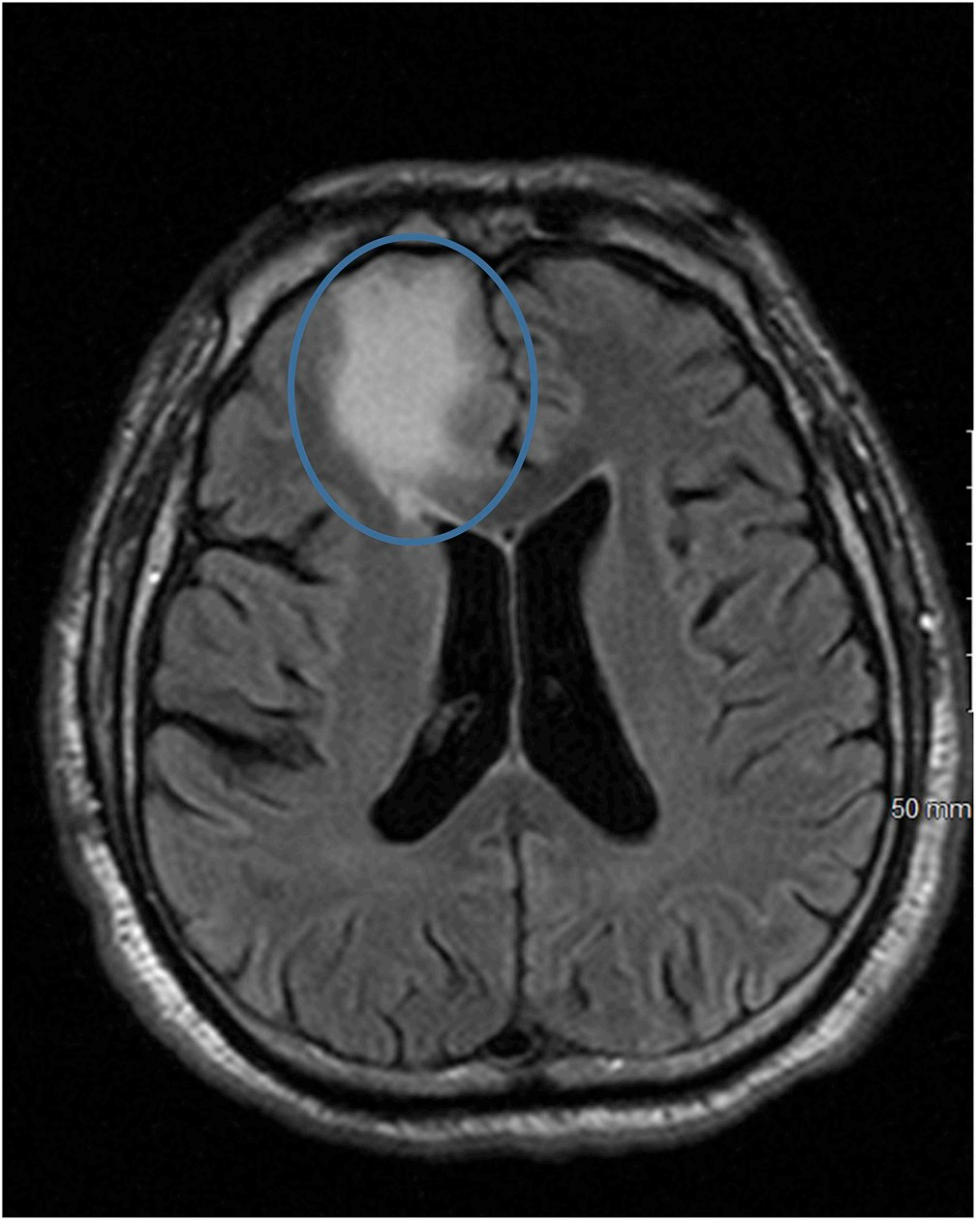
Axial flair sequence of the brain showing vasogenic edema present within the inferomedial right frontal lobe.

**Fig. 3:**
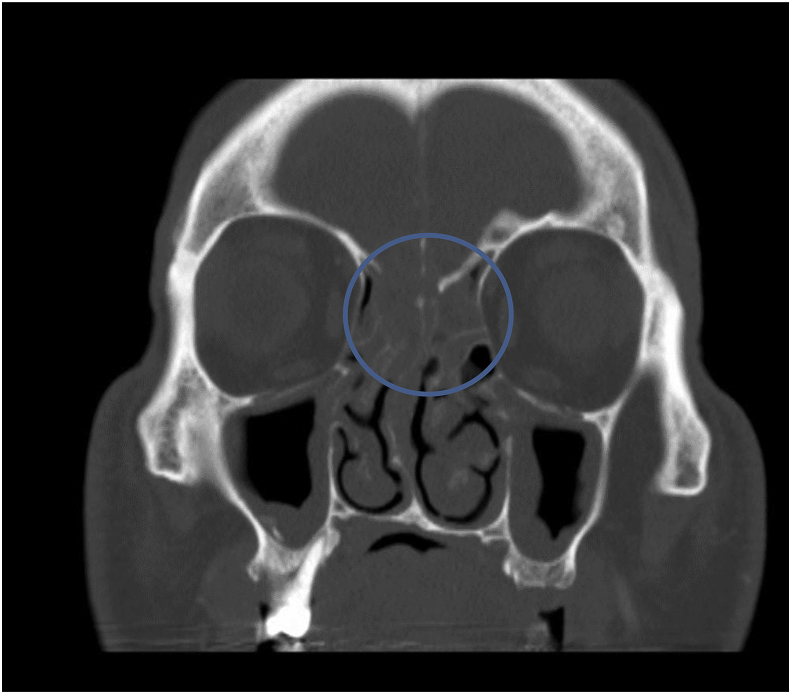
Coronal CT scan of the sinuses showing frontal ethmoidal recesses obstructed bilaterally. Moderate opacification of the ethmoid air cells bilaterally is present. Right sphenoid sinus is hypoplastic and moderately opacified. The right sphenoethmoidal recess is obstructed. Complete obstruction of the ostiomeatal units is present bilaterally due to mucosal thickening.

**Fig. 4:**
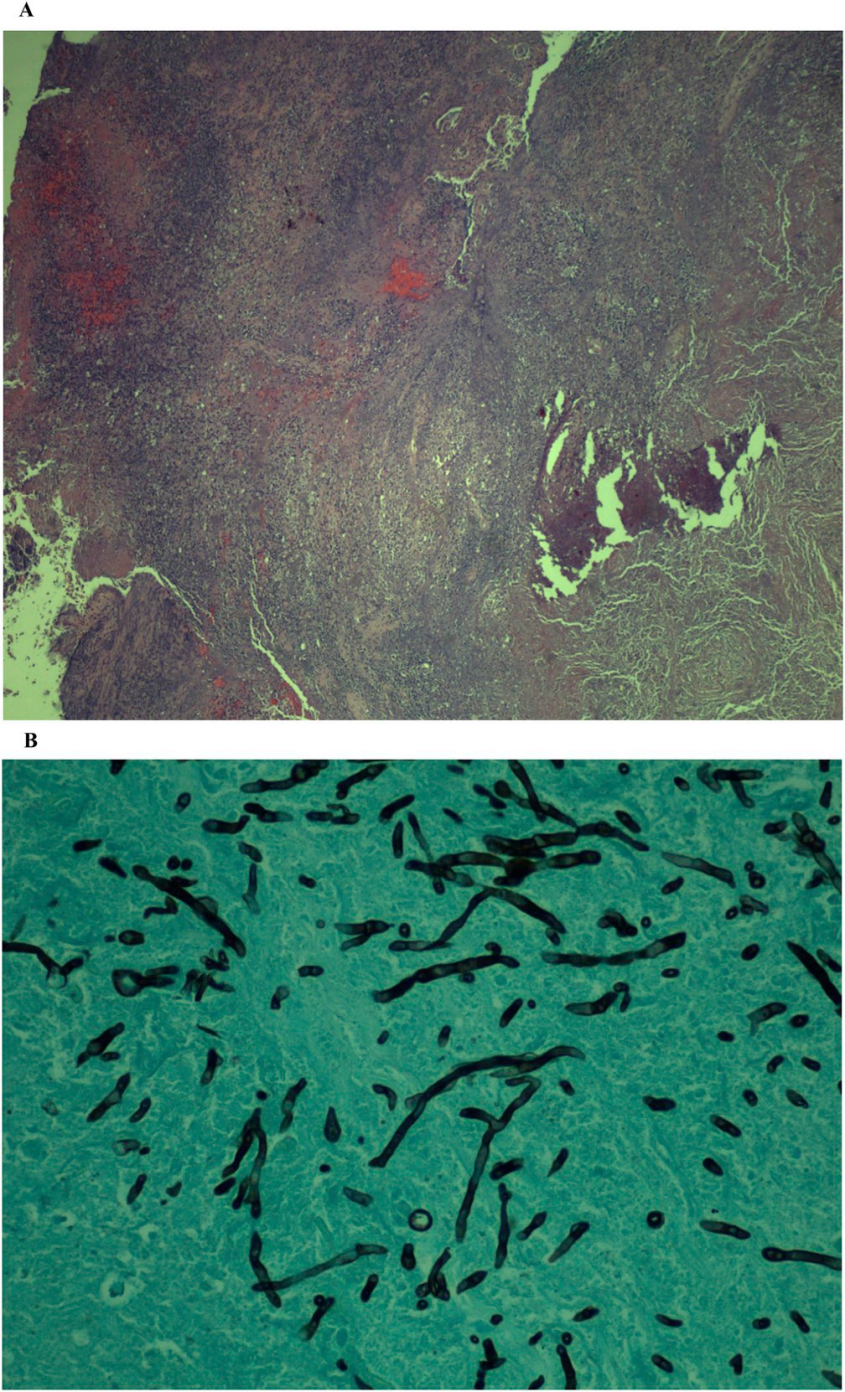
**A.** H&E photomicrograph showing part of brain parenchyma replaced by granuloma comprising of multiple histiocytic giant cells and lymphocytic infiltrates compatible with inflammation. **B.** Grocott's methenamine silver stain of fungal hyphae showing branching at acute angle consisting with *Aspergillus* species.

Computed tomography (CT) scan of the head performed at the day of presentation (Day 0) (Fig. 1) was positive for a decreased attenuation lesion in the right inferior frontal lobe, causing mass effect on the third ventricle and frontal horn of the right lateral ventricle suspicious of subacute stroke and neoplasm. Magnetic resonance imaging (MRI) brain performed on day 1 of presentation (Fig. 2) showed a heterogeneously enhancing cystic and solid mass along the cribriform plate and into right frontal lobe. These findings were suspicious of esthesioneuroblastoma or malignant neuroectodermal tumor or direct intracranial spread of a sinonasal infection. CT scan of the sinuses performed on day 1 (Fig. 3) showed an enhancing mass centered within the olfactory recess of the right nasal cavity. An endoscopic biopsy of the sinonasal mass was performed also on day 1. Cribiform plate cultures were positive for *Aspergillus* species 3 days after presentation (Fig. 4A–B). Since the organisms were detected on culture using a Grocott's methenamine silver stain, testing for fungal genes by a PCR was not performed.

The patient tolerated well a bilateral frontal craniotomy, cranialization of frontal sinus, and removal of frontal lesion with debridement of sinus. He was started on intravenous (IV) voriconazole 6 mg/kg twice daily for 1 day then 4 mg/kg twice daily for another 6 days then switched to oral dosing of 200 mg twice daily for 12 weeks. For a period of 1 month after discharge where the he was still on oral voriconazole, no seizures, worsening symptoms, headaches, intermittent vertigo, hearing or vision loss, nausea, congestion, rhinorrhea, cough, chills, or fevers were reported. A follow-up visit after 3 months of his last voriconazole dose showed a cooperative and conscious patient with no generalized complaints.

## 3 Discussion

Primary mycoses of the paranasal sinus contains *Aspergillus, Candida, and Mucor* with *Aspergillus* reported as the major causative organism for the majority of cases [4]. *Aspergillus* species are saprophytes within the environment. *Aspergillus fumigatus* is the most common human pathogen. The lungs are the primary site of infection because *Aspergillus* tends to enter the human body in the form of spores by inhalation [5]. CNS infection may occur through different ways: Hematogenous dissemination from the lungs [2], direct extension from the paranasal sinuses and orbits [6], or direct inoculation at the time of surgery [7]. Our patient used to work as a coal miner and currently works in a transporting grocery store which might predispose him to inhalation of *Aspergillus* spores. Similar to other cases presented in the literature, our patient had diabetes mellitus type 1 as a possible predisposing factor for the development of cerebral aspergillosis.

Whenever cerebral aspergillosis happens, it may present with meningitis, cerebritis, infarction, abscess, granuloma, or mycotic aneurysms. Less commonly, it presents as a space occupying lesion as the clinical presentation of our patient. Table 1 highlights the case reports of cerebral aspergillosis which presented as space occupying lesions in immunocompetent hosts.

**Table 1:** Case reports in the literature identifying cerebral aspergillosis mimicking a space occupying lesion in immunocompetent patients.

Case Report	Patient's Characteristics	Findings	Follow-up
Kim et al. [7]	A 37-year-old woman was admitted due to dull headache which developed 2 months prior to admission. Left cerebellar meningioma was excised 9 months prior to presentation.	CT and MRI showed an irregular-shaped lesion in the left cerebellar hemisphere, the same location of the previous tumor raising suspicion for recurrence.	Mass was surgically excised. Pathology confirmed presence of *Aspergillus* species. Treatment with amphotericin B plus fluorocytosine.
Phuttharak et al. [13]	A 24-year-old man with a recent diagnosis of tuberculosis but not on immunosuppressive regimen presented with severe headaches that were worse on the left side and had persisted a few months before presentation.	CT and MRI of the brain revealed a large isoattenuated left temporoparieto-occipital mass with an irregular hypoattenuated center and surrounding brain edema with calcifications.	A craniotomy and excisional biopsy. Pathological examination confirmed the diagnosis. The patient received long-term aggressive antifungal drug therapy.
Azarpira et al. [14]	The clinical symptoms began one year before admission.	An intracranial granuloma due to *Aspergillus fumigatus* involving the anterior cranial fossa and the frontal lobe that was treated with extensive excision.	Pathology confirmed the diagnosis. Medical antifungal therapy (intravenous amphotericin B) was give, but she failed to respond to these treatments and died.
Kumar et al. [3]	A 17-year-old female presented with generalized acute headache and diminution of vision had a clinical diagnosis of retro-bulbar neuritis.	Contrast enhanced magnetic resonance imaging (CEMRI) revealed a mass along the right planum sphenoidale with extension into the right sphenoid sinus and the anterior pituitary gland.	Endoscopic trans-sphenoid biopsy and curettage was done. Pathology confirmed the diagnosis. Antifungal treatment by intravenous amphotericin was started.
Kumar et al. [3]	A 48-year-old female presented with blurring of vision and right-sided body weakness since 4 weeks. She had right frontal headache for 6 months. On examinations, she had right VI nerve palsy and right body weakness.	The CT and MRI imaging features resembled meningioma; but the bone and cavernous sinus invasion were atypical.	The mass lesion was surgically removed through right temporal approach. Histopathology confirmed the diagnosis of aspergilloma.
Kumar et al. [3]	A 30-year-old young male presented with severe generalized headache, altered sensorium, rhinorrhea, diminishing vision and generalized weakness. On examination, the patient had multiple cranial nerve palsies.	The CT and MRI findings were suggestive of a locally aggressive process possibility representing hematologic malignancy, metastasis or infection possibly fungal or granulomatous in origin.	An endoscopic trans-sphenoidal biopsy was made yielding the diagnosis of *aspergillus* infection.
Kumar et al. [3]	A 50-year-old male presented with right orbital pain and vision disturbance for few months.	The solid T2 hypointense intraorbital intraconal mass simulated a hematologic neoplastic mass (granulocytic sarcoma or lymphoma), especially in association with pachymeningeal enhancement.	Endoscopic trans-sphenoidal biopsy was performed which established the diagnosis of aspergilloma.
Pant et al. [15]	An immunocompetent 34-year-old male	The mass mimicked a meningioma on preoperative imaging.	Not available
Neyaz et al. [16]	A 22-year-old-male presented with recurrent attacks of complex partial seizures with secondary generalization for 1 month, headache and blurring of vision with diplopia for 20 days.	MRI showed a large T2 hypointense mass in the right temporal lobe with intense homogeneous postcontrast enhancement.	Right frontotemporal craniotomy excised the mass. Pathological and microbiological examination confirmed the diagnosis. Amphotericin B and voriconazole were used.

The radiological appearance of CNS aspergillosis is variable depending on the patient's immune status. A thick irregular wall of the mass on CT and MR images indicates a competent host defense mechanism that is attempting to isolate or encapsulate the offending organisms [8]. In immunocompromised patients, imaging of cerebral aspergillosis can reveal multiple ring enhancing lesions, abscess formation, meningitis/meningoencephalitis, and small infarcts with or without hemorrhage due to vasculitis and mycotic aneurysm [[9], [10], [11]]. In immunocompetent individuals, it presents as intracerebral, intracranial-extradural or invading orbit and/or skull base [12].

It is worth mentioning that the good outcome in our case was likely due to the combination of early diagnosis, prompt surgical removal, initiation of aggressive antifungal therapy (voriconazole), and the normal host immune response of the patient.

In conclusion, we have presented a case of cerebral aspergillosis occurring in an immunocompetent individual who presented for sudden onset unresponsiveness. Brain imaging were suspicious for a cerebral mass mimicking a neoplasm extending to the nasal cavity. Imaging of the sinuses and biopsy confirmed the diagnosis of cerebral aspergillosis. Surgical excision and aggressive antifungal treatment yielded a good response.

## Conflict of interest

There are none.
